# Characterising Upper Limb Movements in Huntington's Disease and the Impact of Restricted Visual Cues

**DOI:** 10.1371/journal.pone.0133709

**Published:** 2015-08-06

**Authors:** Jessica Despard, Anne-Marie Ternes, Bleydy Dimech-Betancourt, Govinda Poudel, Andrew Churchyard, Nellie Georgiou-Karistianis

**Affiliations:** 1 School of Psychological Sciences, Faculty of Medicine, Nursing and Health Sciences, Monash University, Clayton, Victoria, Australia; 2 Monash Biomedical Imaging, Monash University, Melbourne, Victoria, Australia; 3 Victorian Life Sciences Computation Initiative, Life Sciences Computation Centre, Melbourne, Victoria, Australia; 4 Department of Neurology, Monash Medical Centre, Clayton, Victoria, Australia; University of Toronto, CANADA

## Abstract

**Background:**

Voluntary motor deficits are a common feature in Huntington's disease (HD), characterised by movement slowing and performance inaccuracies. This deficit may be exacerbated when visual cues are restricted.

**Objective:**

To characterize the upper limb motor profile in HD with various levels of difficulty, with and without visual targets.

**Methods:**

Nine premanifest HD (pre-HD), nine early symptomatic HD (symp-HD) and nine matched controls completed a motor task incorporating Fitts' law, a model of human movement enabling the quantification of movement timing, via the manipulation of task difficulty (i.e., target size, and distance between targets). The task required participants to make reciprocal movements under cued and blind conditions. Dwell times (time stationary between movements), speed, accuracy and variability of movements were compared between groups.

**Results:**

Symp-HD showed significantly prolonged and less consistent movement times, compared with controls and pre-HD. Furthermore, movement planning and online control were significantly impaired in symp-HD, compared with controls and pre-HD, evidenced by prolonged dwell times and deceleration times. Speed and accuracy were comparable across groups, suggesting that group differences observed in movement time, variability, dwell time and deceleration time were evident over and above simple performance measures. The presence of cues resulted in greater movement time variability in symp-HD, compared with pre-HD and controls, suggesting that the deficit in movement consistency manifested only in response to targeted movements.

**Conclusions:**

Collectively, these findings provide evidence of a deficiency in both motor planning, particularly in relation to movement timing and online control, which became exacerbated as a function of task difficulty during symp-HD stages. These variables may provide a more sensitive measure of motor dysfunction than speed and/or accuracy alone in symp-HD.

## Introduction

Huntington’s disease (HD) is an autosomal-dominant neurodegenerative disorder caused by the pathological expansion of a CAG trinucleotide repeat, disrupting cognitive, affective and motor functions [1–3]. A major focus of current research is to identify specific deficits early in the disease, in order to develop targeted treatment protocols that may delay disease progression [4]. The premanifest stage of HD (pre-HD) [5] provides an unprecedented opportunity to characterize deficits and ultimately encapsulate sensitive markers of disease progression from premanifest to symptomatic stages for potential use in clinical interventions [4,6,7]. While the focus of recent research has predominantly entailed the use of advanced neuroimaging technologies to track neuronal activity [7–9], their functional relevance has been less well understood [4]. Importantly, it is the behavioural impact at a functional level that renders an individual incapable of living independently Although many studies have characterised motor deficits in HD [10–29], most notably focus on rudimentary measures, such as speed and accuracy, and often comprise of simple finger tapping tasks [11,30]. As such, there is paucity in the literature as to how this translates into more complex motor behaviours.

The basal ganglia are the main site of neurodegeneration in HD and have previously been implicated in motor preparation and planning, thereby regulating the phasic timing of movements [16,31–36]. This considered, underlying dysfunction in the organisation of movement may persist, over and above the observed deficit of speed and accuracy. For this reason, we aimed to characterize the kinematic profile of more complex movement at different stages of disease, to gain a more comprehensive understanding of how motor dysfunction may translate into functional incapacity.

Studies probing the finer kinematic components of movement in healthy individuals have uncovered that goal-directed aiming requires distinct, yet integrated motor components to achieve a targeted end goal [37–40]. Historically, and based on Woodworth’s model [40], goal-directed aiming of movements comprise of two separate actions. The first, a rapid ballistic burst, orients the limb toward a target [37–41], followed by an ‘online’ control phase incorporating visual and proprioceptive feedback to effectively detect and correct for errors in order for the limb to accurately reach the target [26,37–40]. The initial component (measured as time spent accelerating to reach peak speed) is considered a function of motor planning, whereas the latter component (time spent decelerating from peak speed toward target approach) represents online corrective sub-movements [26,37–40]. Moreover, evidence suggests that each component of movement employs differing neuronal networks, with recruitment of prefrontal regions, basal ganglia, anterior cingulate, and inferior parietal regions, during the initial ballistic action, referred to as the planning network [42,43], followed by subsequent recruitment of the sensorimotor cortex, cerebellum and superior parietal regions during the latter corrective phase, referred to as the online control network [42,43]. Taking these regions into account, HD neuropathology may portray a signature motor profile in terms of movement planning and error processing that could provide insight into the failings of their respective underlying neuronal networks.

Despite extensive research characterising the motor disturbance in symp-HD, few studies have addressed motor function during the premanifest stage [11,25,44,45]. Considering a clinical diagnosis relies on the unequivocal presence of involuntary movement [46], pre-HD would not be expected to exhibit overt motor dysfunction. Interestingly, however, neuroimaging findings clearly depict striatal neuronal loss 15 to 20 years prior to symptom onset [8,47,48]. When voluntary movement is complex and challenged there may be some level of functional impairment in pre-HD, which could be assessed by sensitive tools and kinematic measures. Recent support of this notion has come from studies demonstrating significant increased movement variability in pre-HD [11,18,49,50]. The timing component of basal ganglia function not only determines motor planning, but also influences generalised aspects of movement timing [51–53]. In the instance of pre-HD, early striatal degeneration may result in a disruption to the timing of an action, leading to the inability to maintain consistently timed motions [11,18,49,50]. This has been evidenced in both simple finger tapping [11,18], cued sequential tapping [17], and in the more automated action of walking [49,50]. No study has previously investigated different kinematic components of movement during goal directed aiming. Findings from such a study could provide important new insight about how motor processes breakdown (i.e., movement planning) during the pre-HD stages.

Accordingly, this study sought to quantify the kinematic profile of movement planning and online control in HD using a computerised reciprocal aiming task implementing Fitts’ law, both with and without the restriction of visual cues. Fitts’ law is a formulated model that predicts movement time (MT) when performing a goal-directed action [54,55], by integrating target traits of size and distance. The robust model implements the mathematical formula MT = *a* + *b* [log_2_ (2*A/W*)], where *A* reflects movement amplitude (determined by distance between targets), and *W* represents target width, with *a* and *b* as constants [54,55]. Movement Time is dependent on the difficulty of the task being performed, requiring the planning system to strategically accommodate the limitations of the motor system, and to consequently adjust temporal parameters of the movement. For example, greater task difficulty entails greater corrective sub-movements, thus resulting in increased movement times [56,57]. Application of this model yields a specified index of difficulty (ID), as a function of movement amplitude (distance between targets) and target size (small or large), determined by the equation log_2_ (2*A/W*). This allows quantification of the speed-accuracy trade-off as a function of task difficulty [54,55], with smaller targets at a greater distance placing the greatest demand on the perceptual-motor system. Whilst Fitts’ law has been utilized in other clinical populations [58–62], never before has it been investigated in HD. No previous study, to our knowledge, has investigated the breakdown of movement kinematics in HD to assess the individual contribution of motor planning, separate from error feedback. Moreover, considering the reliance on external cueing due to basal ganglia pathology [13,16,25], there is limited research into how the restriction of visual cues may impact motor performance. Implementing such a task will provide a more comprehensive profile of the underlying motor dysfunction observed in HD across multiple domains.

The aims of the current study are three-fold. Firstly, to determine whether pre-HD and symp-HD groups adhere to Fitts’ law during a reciprocal aiming task; secondly, to determine whether the Fitts’ task is sensitive to detecting subtle movement irregularities, differentiating pre-HD and symp-HD groups from controls; and, finally to investigate whether the absence of visual cues impacts the motor profile of pre-HD and symp-HD groups, compared with controls. In light of HD neuropathology, it is hypothesized that all groups will adhere to Fitts’ law, showing a linear relationship with prolonged movement times as a function of increasing task difficulty, with this effect increasing with greater disease severity. Furthermore, given the role of the basal ganglia in motor planning and coordination of timing [16,31–34], it is expected that symp-HD will show greater impairment in measures of motor planning and of movement variability, compared with controls, under conditions of increasing task difficulty. Given the more profound global deficit in symp-HD, it is also hypothesized that this group will show greater impairment in online control than both controls and pre-HD, and as a function of increasing task difficulty. Moreover, it is hypothesized that the pre-HD group will show some impairment in measures of motor planning and coordination of timing, compared with controls, however the extent of this is unclear. Furthermore, the absence of visual cues is expected to have a more detrimental effect on both HD groups, with symp-HD and pre-HD performing poorer than controls when visual cues are restricted.

## Method

### Participants

Twenty-seven right handed participants (Edinburgh Handedness Test; [63]) aged 40–70 years were recruited for this study. Participants included 9 pre-HD (2 males) and 9 symp-HD (5 males), recruited through the Georgiou-Karistianis Experimental Neuropsychology Research Unit-Stout database, Monash University (Ethics Number 2003/847). Controls (2 males) were matched for age, gender and premorbid IQ (National Adult Reading Test 2^nd^ edition, NART-2; [64]) to the pre-HD group. One-way ANOVAs revealed no significant group differences in estimated IQ (*p* < .05), although one participant failed to perform this task. Naturally, the symp-HD group were significantly older than both the pre-HD and control groups (*p* = .003).

All participants underwent a screening process prior to recruitment, with exclusion criteria including history of neurological or psychiatric disturbance, aside from HD, clinical diagnosis of depression (Beck Depression Inventory—II, BDI-II; [65]), or previous traumatic brain injury. A one-way ANOVA revealed no significant group differences in BDI-II (*p* < .05). Pre-HD and symp-HD participants underwent gene testing prior to enrolment to the study, and CAG repeat length ranged from 39 to 47. All pre-HD and symp-HD were assessed by a collaborating neurologist (Dr Andrew Churchyard) via the use of the Unified Huntington’s Disease Rating Scale total motor score (UHDRS-TMS; [66]). Inclusion in the pre-HD group was based on UHDRS-TMS ≤5 and the symp-HD group based on UHDRS-TMS > 5, as per previous studies [67]. Disease burden score (DBS) [68] was calculated across both groups and estimated years to onset (YTO) of symptoms was calculated for the pre-HD group only [69]. Demographic and clinical data for all groups are provided in Table 1. Symp-HD participants remained on their normal medication regime and reported taking selective serotonin reuptake inhibitor (SSRI) antidepressants (n = 3), neuroleptic medications (n = 3) and Benzodiazepines (n = 1). Neither control or pre-HD groups reported taking any prescribed medications.

**Table 1:** Demographic and neurocognitive data across groups. Means and standard deviations (SD) provided.

	Control (*N* = 9)	Pre-HD (*N* = 9)	Symp-HD (*N* = 9)
	M±SD	M±SD	M±SD
**Gender (M:F)**	2:7	2:7	5:4
**Age (range)**	40.3±10.5 (25–56)	38.2±8.5 (25–50)	54.9±10.1 (43–69)
**IQ (NART)**	114.6±4.8	115.1±7.8	114.9±8.7
**BDI-II**	1.9±1.5	4.5±4.6	6.5±8.3
**CAG repeats**	-	41.9±1.7	43.6±1.4
**UHDRS-TMS**	-	0±0	16.3±5.7
**DBS**	-	238.4±68.1	434.2±63.4
**YTO**	-	20.8±9.5	-
YSO	-	-	6.2±2.8

Note: IQ (NART, National Adult Reading Test); CAG, cytosine-adenine-guanine (number of repeats >40 is full penetrance); UHDRS-TMS, Unified Huntington’s Disease Rating Scale-Total Motor Score (pre-HD, UHDRS-TMS≤5; symp-HD, UHDRS-TMS>5); DBS, Disease Burden Score (CAG-35.5) x age; YTO, Years to onset—estimation expressed as a countdown from current age to 0 = disease onset. YSO, Years since onset of symptoms.

Ethics approval was granted by the Monash University Standing Committee on Ethics in Research Involving Humans, and all participants gave informed consent prior to participation in accordance with the declaration of Helsinki.

### Procedures

The *Fitts’ Task* is a computerized paradigm implemented to analyze movement kinematics. Using custom made software, target stimuli were presented on a 17 inch LCD touchscreen computer (Micro Touch 3M M170, Methuen, MA, USA), positioned centrally to the participant, at a distance of approximately 30cm. Performance was recorded via contact of an interactive stylus upon screen surface, measured at 1 kHz, which was transmitted to a connected laptop. No filter was applied to the pen trace at pre-processing. Kinematic analysis was performed using Matlab software (Version 8.01, MathWorks, MA, USA).

**Fig 1:**
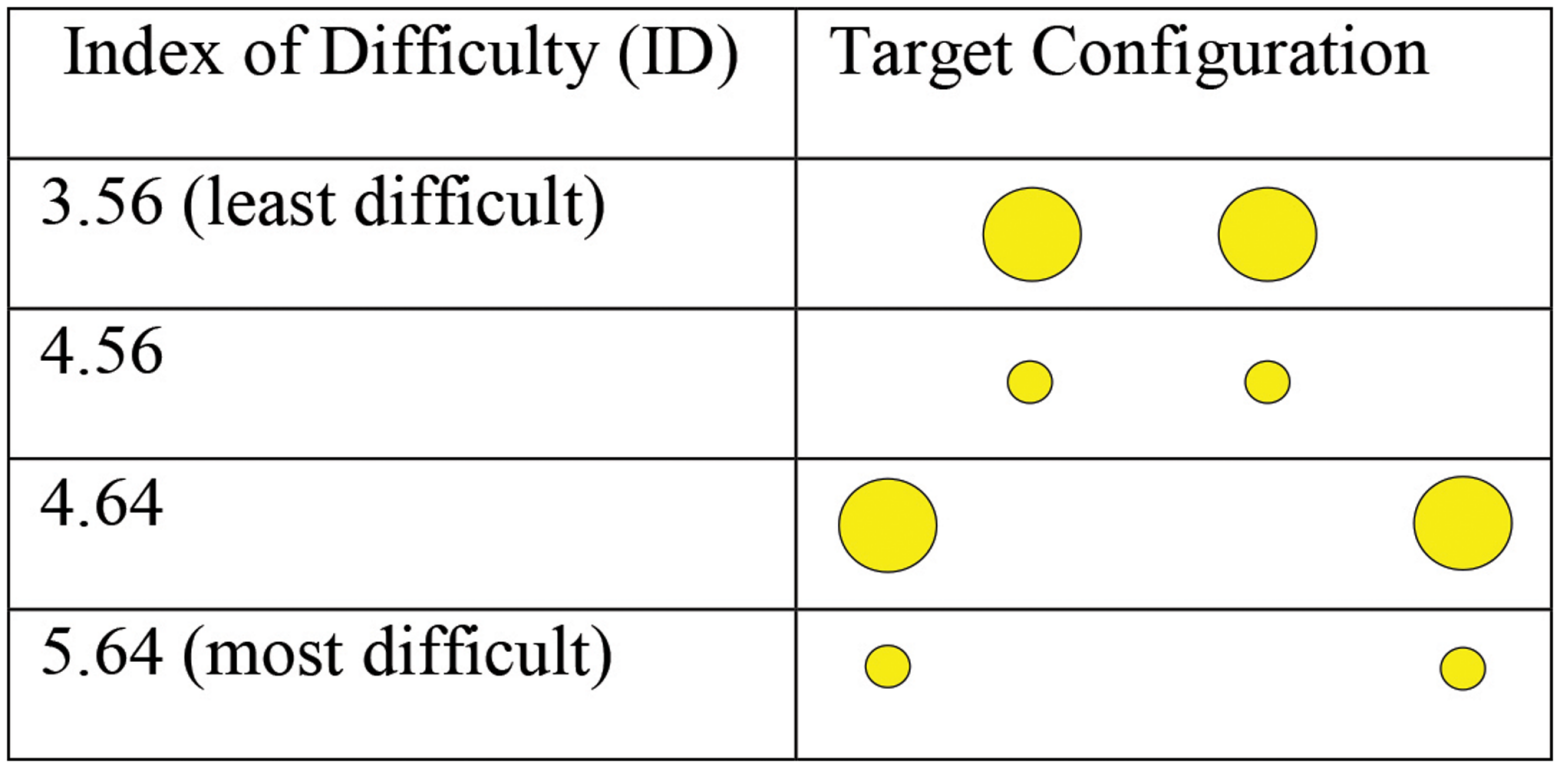
An illustration of the four configurations representing the four conditions and retrospective indices of difficulty.

Two circular yellow targets were presented upon a white background. In each condition target size and distance varied, comprising two levels of target size (large 2cm, or small, 1cm), and two levels of distance between targets (near 12cm, or far 25cm). The combination of size and distance resulted in four trial conditions with corresponding ID’s (see Fig 1).

All participants were instructed to draw reciprocal lines between the two target stimuli using a pen-shaped stylus. Each trial was initiated by stylus contact, and terminated upon stylus removal. Participants completed two practice trials prior to data collection. Using their dominant (right) hand beginning in the centre of the left target, participants performed reciprocal movements between the centre of each circle. After 10 movements, the targets disappeared and participants continued a further 10 reciprocal movements based on their recollection of target location. This allowed for trials with visual cues (initial 10 movements) and trials with no visual cues (subsequent 10 movements) to be statistically analysed. The trial concluded when the word “STOP” appeared on screen. Visual feedback was only provided upon completion of each trial, via a red line highlighting their movement path. A total of 5 trials were performed across each of the four conditions, presented in a pseudo-random order. Participants were instructed not to rest their elbow on the desk (to provide an accurate representation of full upper limb kinematics), not to prematurely remove the stylus (as this would void the trial), and not to count movements (to avoid metronomic influences of counting). Participants were advised to place equal emphasis on both speed and accuracy of movements.

### Statistical analysis

The departure from zero velocity signified movement onset, and return to zero velocity marked the completion of that movement. Each trial was visually inspected to confirm validity, with individual movements identified and analysed via a custom script written using Matlab software (Version 8.01, MathWorks, MA, USA).

The relevant dependent variables obtained from kinematic analysis included: overall movement time, defined as the time from movement onset to movement offset (ms); dwell time, defined as the time spent stationary between offset of the previous movement and onset of the next subsequent movement (ms); peak velocity, the peak speed reached during the movement (cm/ms); mean velocity, the average speed during each trial (cm/ms); time to peak, time taken from movement initiation to reach peak velocity (ms); time after peak, time taken to decelerate from peak speed during target approach and to return to zero velocity (ms); Movement time variability, the standard deviation of individual movement times; Asymmetry index, proportion of time spent accelerating compared with time spent decelerating, (calculated as Time To Peak divided by Movement Time) [70]. Asymmetry Index scores ranged from 0.0–0.9, with a score of 0.5 indicating equal time spent accelerating and decelerating. Accuracy was also considered via measures of constant error (mean distance from target centre in cm) and absolute variable error (standard deviation of movement endpoint in cm). Dependent variables were averaged for each trial, and each cue condition (with or without visual targets separately), and a total average was obtained for each participant. Given that index of difficulty is predicted to influence each of the dependent variables in a systematic way, we will not discuss main effects of index of difficulty. We will report only main effects and interactions involving Group.

A linear regression of Movement time, as a function of Index of difficulty, was performed to address the initial hypothesis, whether each group would display kinematic profiles that conform to Fitts’ law. To explore group differences, three-way mixed model ANCOVA’s, using factors Group (control, pre-HD and symp-HD), ID (3.58, 4.58, 4.64 and 5.64) and Cue Condition (with or without visual targets) were performed across each of the dependent variables whilst covarying for age. Where required, post-hoc one-way ANCOVA’s were performed to further investigate significant interactions. Due to expected age differences between groups, age was used as a covariate across all analyses.

Using Pearson correlation coefficients, data were further analyzed for correlations between significant kinematic measures and clinical parameters. These clinical characteristics included CAG repeat length, UHDRS-TMS and time in years to/since onset. The pre-HD and symp-HD groups were combined to create a new variable, investigating disease characteristics as a continuum [45].

The raw data collected in this study can be viewed in S1 Dataset.

## Results

Preliminary analyses were performed across all data addressing violations, with relevant statistics reported below. The following section presents the results according to variables of interest.

### Movement time

**Fig 2:**
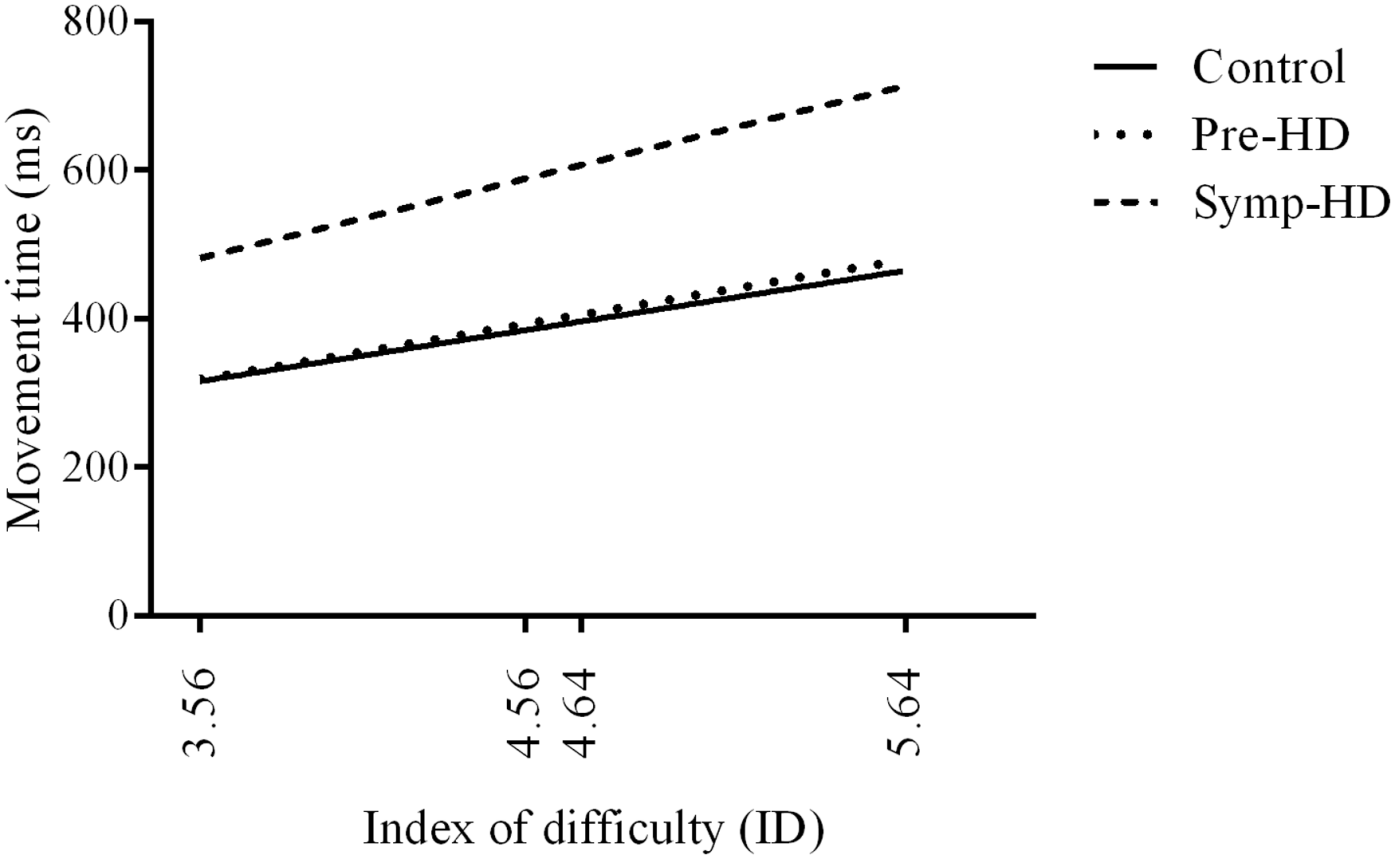
Linear regression of movement times as a function of index of difficulty.

All participants conformed to Fitts’ law, with a linear regression analysis revealing a positive relationship between Movement time and Index of difficulty (Fig 2). All groups expressed a moderate fit to the regression model (controls R^2^ = .63, *p* = .029); pre-HD R^2^ = .50, *p* = .016; symp-HD R^2^ = .52, *p* = .005). A one-way ANCOVA was performed on the slope of regression lines for each participant, which revealed no significant Group difference *F*(2,26) = 1.68, *p* = .207. For all groups, Movement time increased as a function of increasing Index of difficulty.

**Fig 3:**
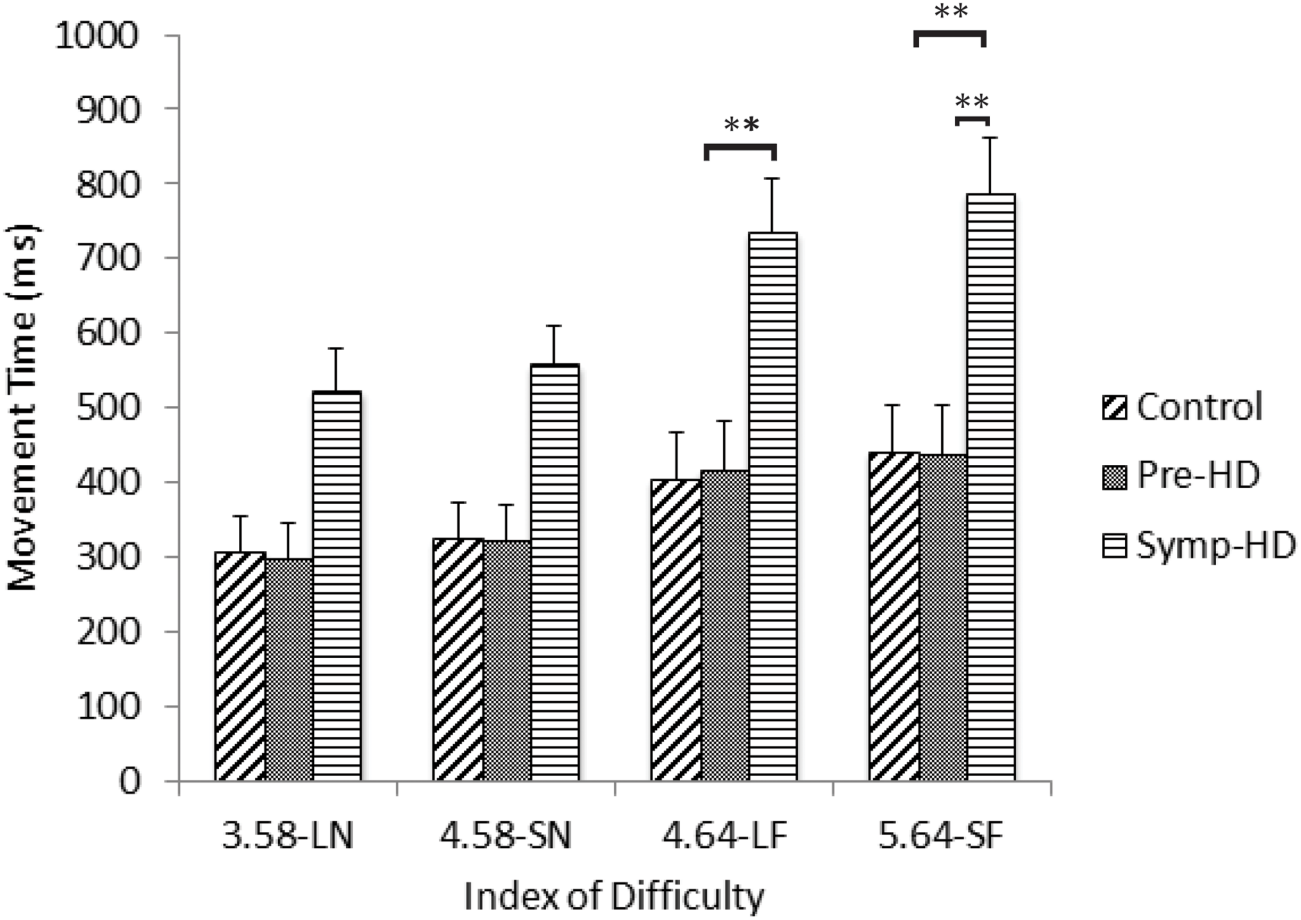
Movement time between groups as a function of index of difficulty. Standard error bars included. Note: LN = large near, SN = small near, LF = large far, SF = small far. ** = p < .01.

A three-way ANCOVA revealed no significant main effect of Group. There was a significant Group by ID interaction, *F*(3.56, 39.13) = 3.56, *p* < .020, η_p_^2^ = .239. After Bonferroni corrections, post-hoc ANCOVA’s revealed that the symp-HD group (4.64-LF: *M* = 677, *SE* = 419; 5.64-SF: *M* = 716, *SE* = 433) performed significantly (*p* < .008) slower than controls at the two highest ID conditions (4.64-LF: *M* = 429, *SE* = 103; 5.64-SF: *M* = 469, *SE* = 109), and significantly (*p* < .008) slower than pre-HD (5.64-SF: *M* = 481, *SE* = 126) only at the highest ID condition (Fig 3). There were no other significant interactions.

### Dwell Time

**Fig 4:**
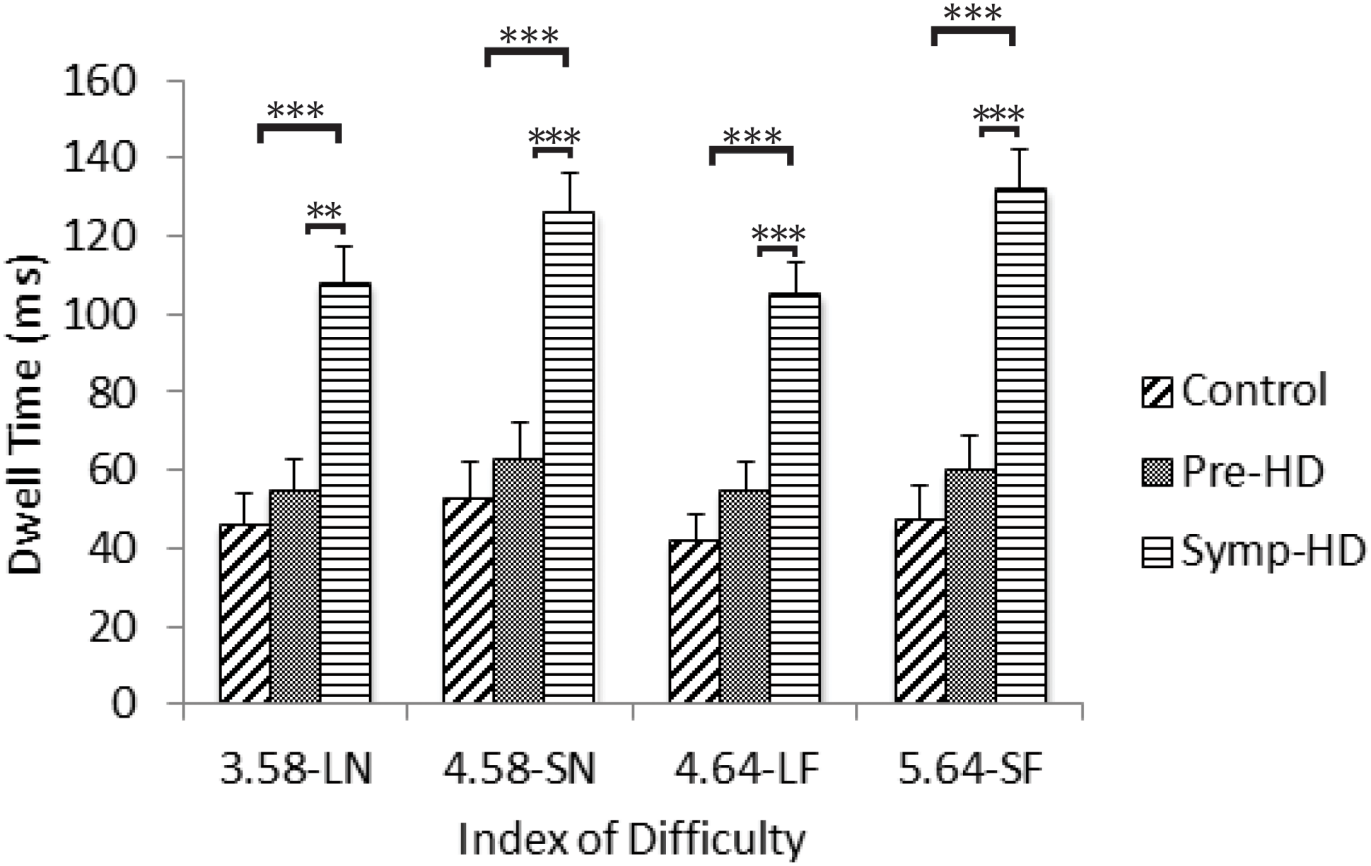
Dwell times between groups as a function of index of difficulty. Standard error bars included. Note: LN = large near, SN = small near, LF = large far, SF = small far. ** = p < .01, *** = p < .001.

A three-way ANCOVA revealed a significant main effect of Group, *F*(2, 23) = 8.36, *p* = .002, η_p_^2^ = .421; the symp-HD group (*M* = 105, *SE* = 17) expressed significantly longer dwell times than both the control (*M* = 53, *SE* = 6.45; *p* = .002) and pre-HD (*M* = 66, *SE* = 9; *p* = .011) groups. A significant Group by ID interaction was also found, *F*(5.71, 65.66) = 2.47, *p* < .05, η_p_^2^ = .177. After Bonferroni corrections, post-hoc ANCOVA’s revealed that the symp-HD group (3.58-LN: *M* = 95, *SE* = 17; 4.58-SN: *M* = 113, *SE* = 18; 4.64-LF: *M* = 94, *SE* = 14; 5.64 = SF: *M* = 116, *SE* = 19) presented with significantly (*p* < .001) longer dwell times than both the control group (3.58-LN: *M* = 51, *SE* = 6; 4.58-SN: *M* = 57, *SE* = 7; 4.64-LF: *M* = 47, *SE* = 6; 5.64 = SF: *M* = 53, *SE* = 7) and the pre-HD group (3.58-LN: *M* = 63, *SE* = 8; 4.58-SN: *M* = 70, *SE* = 9; 4.64-LF: *M* = 62, *SE* = 7; 5.64 = SF: *M* = 70, *SE* = 10) across each index of difficulty (Fig 4). There were no other significant interactions.

### Peak Velocity and Mean Velocity

A three-way ANCOVA revealed no significant Group effects for mean velocity *F*(2, 23) = 0.68, *p* = .52 or peak velocity *F*(2, 23) = 0.27, *p* = .76. There were no significant interaction effects for mean velocity or peak velocity.

### Time To Peak Velocity

A three-way ANCOVA revealed no significant Group effects, *F*(2, 23) = 2.11, *p* = .14, or interactions in time to peak velocity.

### Time After Peak Velocity

**Fig 5:**
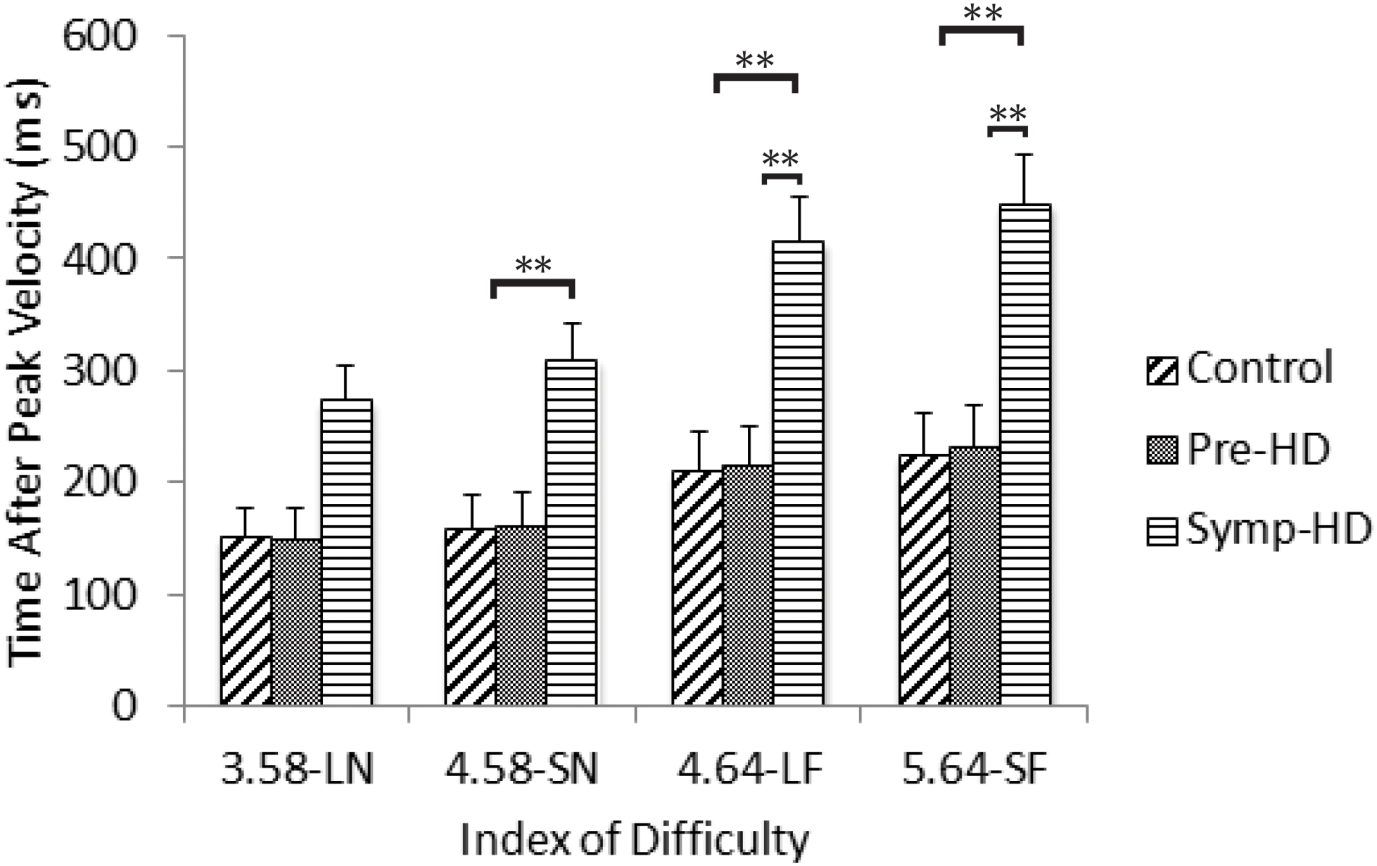
Time after peak velocity between groups as a function of index of difficulty. Standard error bars included. Note: LN = large near, SN = small near, LF = large far, SF = small far. ** = p < .01.

A three-way ANCOVA revealed no significant main effect of Group *F*(2, 23) = 3.22, *p* = .058. There was a significant Group by ID interaction, *F*(4.02, 46.3) = 4.24, *p* = .005, η_p_^2^ = .270. Post-hoc ANCOVA’s revealed that the symp-HD group (4.58-SN: *M* = 282, *SE* = 66; 4.64-LF: *M* = 377, *SE* = 81; 5.64 = SF: *M* = 402, *SE* = 84) took significantly (*p* < .008) longer to decelerate from peak speed than controls at the three higher ID conditions (4.58-SN: *M* = 170, *SE* = 15; 4.64-LF: *M* = 224, *SE* = 21; 5.64 = SF: *M* = 243, *SE* = 23), and significantly (*p* < .008) longer to decelerate from peak speed than pre-HD (5.64 = SF: *M* = 258, *SE* = 28) only at the two highest ID conditions (Fig 5). There were no other significant interactions.

### Movement Time Variability

**Fig 6:**
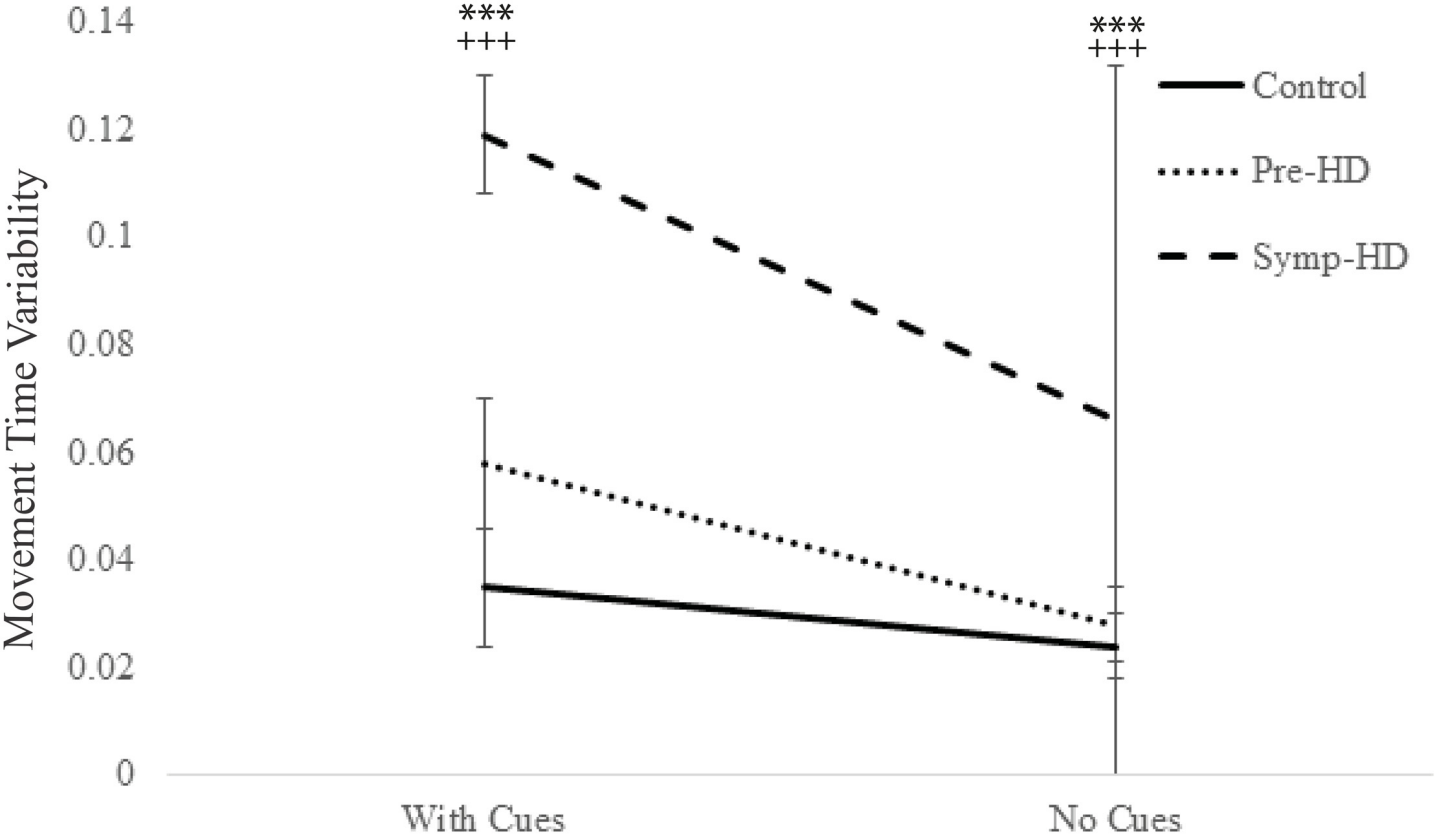
Movement time variability between groups as a function of cue condition. Standard error bars included. *** = p < .001, between symp-HD and controls; ^+++^ = p < .001, between symp-HD and pre-HD.

A three-way ANCOVA revealed a significant main effect of Group, *F*(2, 23) = 11.87, *p* < .001, η_p_^2^ = .508; the symp-HD group (*M* = .093, *SE* = .009) performed with significantly greater variability than both the control (*M* = .043, *SE* = .008; *p* < .001) and pre-HD (*M* = .043, *SE* = .008; *p* = .004) groups. There was also significant main effect of Cue condition, *F*(2, 23) = 66.12, *p* < .001, η_p_^2^ = .742; all groups showing significantly greater variability when cues were present (*M* = .071, *SE* = .006; *p* < .001), compared with when they were removed (*M* = .039, *SE* = .003; *p* < .001). Additionally, significant Group by Cue condition interaction was found, *F*(4.71, 23) = 7.13, *p* = .008, η_p_^2^ = .383. After Bonferroni corrections, post-hoc ANCOVA’s revealed that the symp-HD group (Cues: *M* = .12, *SE* = .007; No Cues: *M* = .066, *SE* = .004) displayed significantly (*p* < .001) greater variability than both the control (Cues: *M* = .035, *SE* = .006; No Cues: *M* = .024, *SE* = .004) and pre-HD groups (Cues: *M* = .058, *SE* = .006; No Cues: *M* = .028, *SE* = .004), under both cue conditions (Fig 6). In addition, the pre-HD group showed greater variability than controls, at a significance of *p* = .027, just shy of the Bonferroni adjusted value of *p* = .017 to be statistically significant. There were no other significant interactions.

### Asymmetry Index

A three-way ANCOVA revealed no significant Group effects, *F*(2, 23) = 2.59, *p* = .097, or interactions in asymmetry index.

### Constant Error

A three-way ANCOVA revealed no significant Group effects, *F*(2, 23) = 0.63, *p* = .54, or interactions in constant error.

### Variable Error

A three-way ANCOVA revealed a significant main effect of Cue Condition, *F*(1, 23) = 7.32, *p* = .013, η_p_^2^ = .241; all individuals showed significantly (*p* < .001) greater variability in movement end point in the cue condition without visual targets (*M* = .90, *SE* = .04), than the cue condition with visual targets (*M* = .62, *SE* = .05). No further significant Group effects, *F*(2, 23) = 2.68, *p* = .09, or interactions were found.

### Correlations

Pearson’s correlation coefficients were used to assess the relationship between motor measures that differentiated between groups (movement time, dwell time, time after peak and movement time variability) and HD clinical disease characteristics. These correlations are represented in Table 2.

**Table 2:** Pearson correlations of significant variables with clinical measures.

	Movement Time	Dwell Time	Time After Peak	Movement Time Variability
**CAG**	.18 (.032)	.26 (.002)	.19 (.020)	.34 (< .001)
**UHDRS-TMS**	.50 (< .001)	.47 (< .001)	.49 (< .001)	.54 (< .001)
**DBS**	.16(.058)	.21(.011)	.17(.036)	.42 (< .001)
**YTO/YSO**	.31 (< .001)	.27(.001)	.32 (< .001)	.42 (< .001)

Note. Pre-HD and symp-HD were combined to create a new variable to explore the relationship between disease characteristics as a continuum. CAG, cytosine-adenine-guanine repeat length; UHDRS-TMS, Unified Huntington’s Disease Rating Scale total motor score; DBS, Disease Burden Score; YTO/YSO, years to onset/ years since onset, expressed as a continuum, pre-HD counting down to 0, symp-HD counting up from 0. r = .10 to .29 small correlation, r = .30 to .49 moderate correlation, r = .50 to 1.0 large correlation (Pallant, 2011). Figures in parentheses are p values.

As presented in Table 2, movement time variability showed the strongest correlations, with moderate to large positive associations across all clinical measures. Dwell time and time after peak showed small to moderate positive correlations across all clinical measures. Movement time showed small to moderate correlations with only years to/since diagnosis, CAG repeat length and UHDRS-TMS.

## Discussion

The aim of this study was to characterise motor performance in pre-HD and symp-HD, compared with controls, in accordance with Fitts’ law, to determine whether the Fitts’ task is sensitive to subtle movement irregularities across groups, and to determine whether any notable irregularities were exacerbated in the absence of visual cues. In support of the first hypothesis, all groups conformed to Fitts’ law, demonstrating increased movement times with corresponding increments in index of difficulty. Although no significant group differences were found on the slope of regression lines, the significant Group by ID interaction showed that symp-HD performed slower as a function of greater task difficulty, compared with pre-HD and controls. The second hypothesis was partially supported; symp-HD dedicated more time to movement planning, as evident by increased dwell times compared with controls, although no group differences were observed in time spent accelerating to peak speed. Symp-HD also showed significantly greater variability in movement times compared with both pre-HD and control groups. Although pre-HD showed a trend towards increased dwell times, their performance on the planning variables (dwell time and time to peak) did not differ significantly from controls. They did however show greater variability in movement times than controls; however, this was not statistically significant after Bonferroni corrections. The third hypothesis was also supported; symp-HD demonstrating a reduced capacity for online control, as evidenced by the excessive time spent decelerating from peak speed compared with both controls and pre-HD. Contrary to the fourth hypothesis, group differences were apparent, but not in the direction expected, and were only observed in the measure of movement time variability. Greater variability was apparent in the presence of visual cues compared to their absence, which was shown to increase with disease severity.

The extended movement times observed in the symp-HD group, which were exacerbated as a function of increased task difficulty, supports previous literature [11,16,71,72]. Interestingly, although movement times increased in symp-HD, there were no group differences in speed of movement as measured by both peak and mean velocities. This suggests that increased movement time was not attributable to overall slowness in symp-HD, who performed comparably to pre-HD and controls. One suggestion for this disconnect relates to movement trajectory. For example Carella et al. [13] also found that symp-HD performed at a comparable velocity to controls, during a square tracing task, albeit whilst travelling significantly greater distances; the authors attributed this finding to more curvilinear trajectories, thought to arise from an increase in multiple sub-movements by the HD group during the trace [13]. Sub-movements are thought to represent a directional change [26,72,73], implying that the individual has detected an error in trajectory, and subsequently has corrected for it by altering their path. This notion is supported by the fact that such sub-movements predominantly occur at the end stages of movement [37–39,74]. With prolonged times spent after peak velocity, our findings provide further evidence that goal-directed movements in symp-HD are laden with late stage jerks, attributable to defective online control. Researchers have postulated an inadequacy in feedback control mechanisms in symp-HD, arising from an inability to effectively integrate sensorimotor information [75]. In this case however, a deficiency in sensorimotor integration would likely be reflected in extended deceleration times across all ID conditions. With extended deceleration times apparent, as a function of increasing task difficulty, our findings instead, support the theory of a compromised internal switch, with symp-HD unable to effectively disengage from their current action. Assuming this is the case, symp-HD may perseverate with each successive movement, resulting in an exacerbated trajectory along an undesired path prior to correction, making multiple corrective sub-movements necessary.

While it is apparent that there is a deficit in online control in symp-HD, motor planning appears to also be impaired, with dwell time showing significant group differences across all ID levels. Interestingly, however, no group differences were observed in time to peak, thought also to be a function of motor planning [38–40]. The contrast in findings, across the two measures, may indicate that each variable is measuring a different motor component. Since the basal ganglia plays a role in the planning of movement and in particular to direct when to instigate or terminate an action [76], it is possible that the measure of dwell time is representing a feature of the planned timing of a motor action. In contrast, the time taken to reach peak speed may in fact be a reflection of the quality of the motor plan [71], incorporating a more proprioceptive representation of the plan, including factors such as movement amplitude and force [77]. It is possible that the motor plan itself may not be defective in symp-HD; rather it could be that the planned timing of movement initiation is disrupted.

Further supporting this theory is the increased variability of movement times expressed by symp-HD, and to some extent, pre-HD. This supports previous findings of a disruption in the coordination of movement timing early in HD, which may be a sensitive preclinical marker of disease progression [11,18,49,50]. Interestingly, however, these differences were most notable in the cued condition. The timing mechanism of the basal ganglia initiates an internal cue, providing context as to when an action should begin and end [36,78]. Efficient movements rely on both internal and external cueing; however, in the instance of HD where the basal ganglia, and subsequent internal cueing mechanism is compromised, the external cueing system must actively compensate [31,35]. Individuals with HD were expected to show greater reliance on the provision of external cues to guide motor performance as evidenced from previous studies [13,17,25]; however, the current findings did not support this. Movement time consistency was reduced in the cued condition, suggesting the absence of visual cues unexpectedly enhanced performance. It has been suggested that removing visual cues, thus removing the target objective, results in a simple aimless action where accuracy is trivial [74,79,80]. The removal of cues likely simplified the task, modifying the movement into an automated action. Therefore, the need to recruit fronto-striatal circuitry (preferentially reserved for novel behaviours [81]) was reduced, thus minimising the impact this dysfunctional circuitry could have on behavioural outcomes. Whilst findings were inconsistent in the absence of visual cues, the cued condition did support previous findings, showing a marked disruption in the consistency of movement timing in symp-HD. Moreover, movement variability demonstrated consistent moderate to strong correlations across all clinical measures, suggesting that movement time variability alongside dwell time and time after peak are perhaps more sensitive measures of the voluntary movement deficit in HD, than simple measures of movement time, speed or accuracy.

Although no group differences were observed between the pre-HD group and controls, clear trends were observed in the measures of movement time variability and dwell time, providing justification to further explore more subtle motor measures across disease stages. Furthermore, the pre-HD cohort was relatively young and substantially far from onset (mean 20.8 years to onset), which may have masked the significance of any subtle motor deficits. Furthermore, when interpreting our results it is important to consider that the symp-HD group was significantly older than the pre-HD and control groups. This poses a limitation for assessing differences between symp-HD and controls, despite the fact that age was included as a covariate in all analyses. The relatively small sample size in each group may have rendered the analysis vulnerable to type II statistical error. Note that care should be taken when interpreting the pattern of results and possible influence of medication. For example of the medications reportedly taken by the symp-HD group, SSRI’s [82], neuroleptic’s [83], and Benzodiazepines [84] have all been reported to improve motor performance, particularly in suppressing chorea, although controversy remains as to the efficacy of these findings [85,86]. Note also, while participants conformed to Fitts’ law, the proportionate increase in ID was relatively minor. These small scale movements may not be entirely representative of actual everyday real-world actions, therefore over interpretation should be avoided.

This is the first study, to our knowledge, that has characterised upper-limb movement in accordance with Fitts’ law in both pre-HD and symp-HD groups. Overall, the voluntary motor deficit depicted in symp-HD is in-line with previous literature, and is consistent with the global neurodegenerative profile observed in symp-HD. Collectively, these findings provide evidence of a deficiency in both motor planning, particularly in relation to movement timing and online control in symp-HD, which is exacerbated as a function of task difficulty. Findings suggest that simple measures of movement time and accuracy may not be sufficient at detecting subtle motor irregularities, and consideration should be made to incorporate alternative kinematic measures such as movement time variability, dwell time and time after peak (shown to be highly sensitive in symp-HD) into the battery of motor assessments implemented in large scale biomarker development studies with larger samples. These findings contribute further to our characterization of motor function in HD and may provide guidance for future interventions to improve the functional capacity of individuals suffering from HD.

## Supporting Information

S1 Dataset File provides the raw data associated with this research. (XLSX)
